# Use of Mobile Forms in Low-Resource Areas for Population Health Surveys: Interview and Field Test Study

**DOI:** 10.2196/53715

**Published:** 2025-08-11

**Authors:** Alexander Davis, Aidan Chen, Milton Chen, James Davis

**Affiliations:** 1 University of California, Santa Cruz, CA, 95064, United States; 2 VSee Health, Newton, MA, 02458, United States; 3 Department of Computer Science and Engineering, University of California, 1156 High St, MS:SOE3, Santa Cruz, CA, 95064, United States, 1 (831) 459 1841

**Keywords:** mobile forms, offline forms, electronic data capture, design, low-resource settings, health surveys

## Abstract

**Background:**

Population health surveys are an important tool to effectively allocate limited resources in low-resource communities. In such an environment, surveys are often done by the local population with pen and paper. Data thus collected are difficult to tabulate and analyze.

**Objective:**

The objective of this study was to evaluate the viability and efficiency of mobile forms as an alternative to paper-based surveys in a specific low-resource setting.

**Methods:**

We conducted pilot interviews with 53 local surveyors in the Philippines to assess their initial attitudes toward mobile forms. We then built software that can generate mobile forms that are easy to use, capable of working offline, and able to track key metrics such as time to complete questions. Our mobile form was field-tested in 3 locations in the Philippines with 33 surveyors collecting health survey responses from 266 participants.

**Results:**

In the pilot phase, we found that 32 out of 53 (60%) of the local surveyors preferred mobile forms over paper. After field-testing, the number of surveyors preferring mobile forms increased to 25 out of 33 (76%) after just using the form a few times. The mobile forms overall demonstrated enhanced efficiency in data collection and usability over paper surveys.

**Conclusions:**

Our findings indicate that mobile forms are a viable method to conduct large-scale population health surveys in this low-resource environment.

## Introduction

There is an unmet need for medical care in low-resource communities, and telehealth clinics have the potential to reach patients in remote regions with insufficient coverage [1]. Our team and VSee interns have operated a series of free clinics with both in-person and telehealth physicians serving low-income populations in the Philippines with our partner organization Gawad Kalinga. Since Gawad Kalinga builds free housing in 10,000 locations across the Philippines, it can reach over 1 million households and mobilize many volunteers to support health initiatives, therefore letting us expand our ability to conduct large-scale health surveys [2]. Gawad Kalinga expressed interest in opening free clinics in all of its locations. We hypothesized that our mobile forms would be more efficient than traditional paper methods due to the ease of use of our software. Our goal was to evaluate whether mobile forms could reduce time spent in data collection, enhance ease of use, and improve the accuracy of captured data [3].

**Figure 1.**
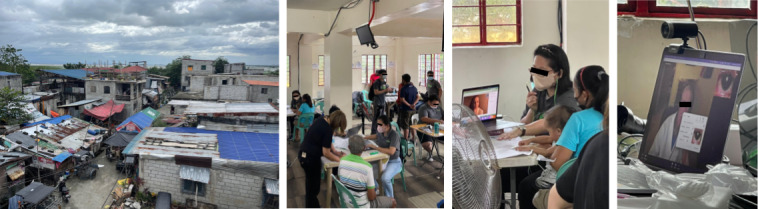
Images from one of our telehealth clinics in Manila, Philippines. The clinics were run by VSee, a US telehealth company. From left to right, the images portray the residential neighborhood in which the clinic took place, the interior of the clinic, a doctor (virtually on the laptop) seeing a patient, and a remote telehealth eye examination.

In order to understand the target population and how best to serve them, we will perform a large set of surveys. Figure 1 provides an example of one community and our free clinic. The figure shows images of a street scene of the community, the interior of one of the health clinics, a remote doctor seeing a patient using telehealth, and a remote eye exam.

A good solution to conduct a large survey in a low-resource community can be challenging. Paper surveys are easy to use and record the data, but they would require a large amount of effort to transcribe information into digital format suitable for analysis [4]. Some health clinics adopt a parallel paper and digital format in an attempt to get the best of both [5]. Many organizations in affluent nations use dedicated tablets or laptops to conduct digital surveys, but in the Philippines, this solution is too expensive, and many people are not comfortable using such digital devices. A good platform solution to address these unique requirements is to use mobile phones already owned by the surveyors to conduct the survey [6].

The research aims to show that surveys conducted on mobile phones are feasible and efficient for data capture in low-resource and remote regions. We conducted pilot interviews to understand user requirements, built survey forms software, ran field-testing on surveys generated by the software, analyzed survey results, and performed postsurvey interviews.

Based on feedback from the pilot interviews, we determined that our mobile forms needed to satisfy 3 conditions. They should be extremely easy to use, allowing surveyors to complete the surveys with minimal training [7]. They should also function properly even if the internet went out. Finally, the survey should be able to measure the time required to type and complete questions. No existing mobile forms satisfied these conditions, so we created software that can generate this type of mobile forms.

A field study was performed to evaluate the practicality of our mobile forms. Over 3 days, in 3 separate locations, 33 surveyors, who were part of an initial group of 53, collected 20-question surveys from 266 respondents from the local population. The final count of 33 surveyors reflects those who were present for the postsurvey interviews, as 20 of the initial surveyors did not make it through the entire process.

After field-testing was complete, we interviewed the surveyors to evaluate user experience and satisfaction.

The contribution of our work is:

- Pilot interviews of surveyors
- Survey forms software: offline, easy, and timing aware
- Field-testing the mobile form–based surveys
- Analysis of field deployment

## Methods

### Pilot Interviews

We had little information on the background of our volunteer surveyors and how much knowledge they had in regards to using a phone, let alone completing a survey on one. A pilot interview to 53 surveyors was conducted to determine how comfortable the surveyors actually were with technology. We did this by manually interviewing each surveyor and asking them questions about preference and experience. Surveyors interviewed were chosen through convenience sampling based on their availability and proximity to survey sites. They were teens to seniors in their 70s, predominantly from low-income communities in Metro Manila.

### Survey Forms Software

To evaluate mobile forms’ performance and improve its design, we need to track information such as how much time surveyors take to complete each question. Therefore, a suitable mobile form must be easy to learn and use, able to work offline, and capable of tracking time spent.

While there are software solutions that can generate mobile forms for low-resource environments, none satisfied all of our needs. For instance, REDCap (Vanderbilt University) and SurveyMonkey (SurveyMonkey) can work offline and track response times, but their complexity or cost are not suitable for our use case, EpiCollect (Oxford University) [8] lacks the ability to track the time data we desired, Google Forms does not function offline, and ODK (University of Washington) [9] was deemed too complex by some stakeholders. We thus developed a software that generates mobile forms that can work offline with data analysis and visualization capabilities.

The mobile form software was developed using Next.js and React for the front end, with Redux for state management. Firestore was used for real-time database functionality and Firebase (Google) handled back-end processes. Offline capabilities were managed through Redux’s offline storage, ensuring data persistence even without internet access, and time-tracking functionality was implemented within the app’s React components. The source code is available on GitHub under an open-source license to allow for replication and adaptation.

A feature of our mobile form is that it can work whether connected to the internet or not. It is common to experience unstable internet in low-resource environments where we collect data. At one of our previous clinics using Google Form, we observed intermittent internet outages to be sufficiently frustrating that some surveyors started writing on paper.

Another feature of our mobile form is that it tracks time data, so that we can study ease of use and quantify how each question will impact total effort required to collect a large number of surveys. The mobile form can measure time spent on each question, time spent typing the answer to each question, time the user spent to complete the survey, and the time that a user is not connected to the internet.

**Figure 2.**
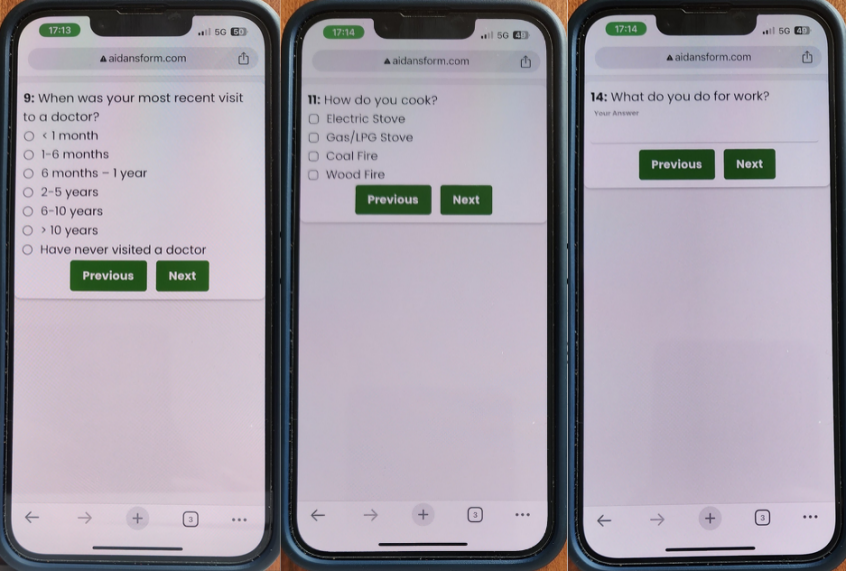
Mobile forms were created to work offline and track the time each question took the surveyors to complete. The interface was designed to be easy to learn and use, with only one question per loaded page.

To encourage surveyors to use phones, we made our mobile form as easy and intuitive as possible. We followed design recommendations for low-resource populations such as minimization of visual complexity and streamlined navigation [10]. Our interface is shown in Figure 2. We presented only one question per screen to minimize scrolling and removed all extra interface buttons that might confuse users. Our goal was to make people feel comfortable using our mobile forms, and they would prefer it over paper.

### Field Test

**Figure 3.**
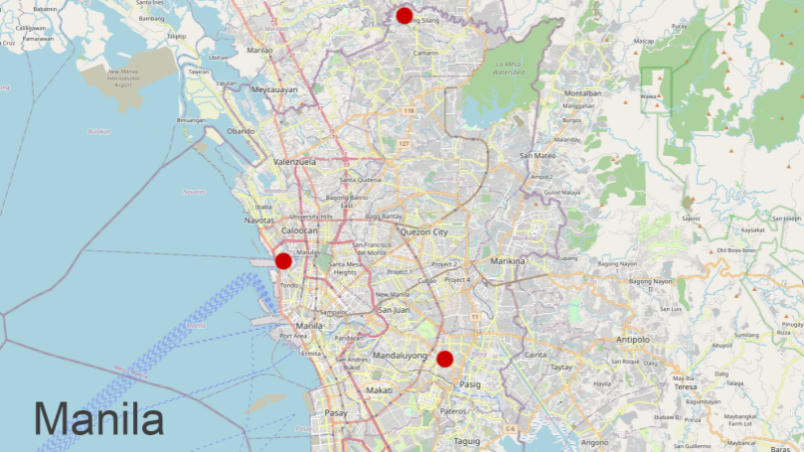
Field tests occurred at 3 metro-Manila sites: 1 in Manila City and 2 in the neighboring communities of Caloocan and Pasig. A total of 33 volunteer surveyors participated, collecting 266 population health surveys.

After designing a mobile form survey using our software, we tested its performance at 3 locations on 3 different days. The locations were in Metro Manila and ranged from an abject squatter settlement next to a trash dump to poor neighborhoods with access to water and electricity. Locations are shown on a map in Figure 3. The communities selected had between 600 and 1200 residents and were chosen to be representative of future survey sites.

We had 6 of our own staff training surveyors over 3 days. These surveyors were volunteers from local communities and selected to cover a wide age range. We were concerned that training might take a long time, but 96% of surveyors completed training in 10 minutes or less. The training covered basic phone usage and navigation of the mobile form.

The surveyors performed a total of 266 patient surveys over 3 days. The survey contained 20 questions related to health and access to health care. Some example questions are “What is the closest medical center or clinic that you would go to?” and “How do you get clean drinking water?” We wanted to find out which kind of questions were faster to complete for surveyors that had limited prior experience with mobile forms. The survey designed by our health team included both text and multiple choice questions.

Surveyors reported no issues with battery life or charging of power banks as the surveys were conducted over relatively short durations. This meant there was no need to provide additional power banks or batteries to ensure surveyors were able to complete their tasks effectively without interruptions.

Finally, we wanted to find out if the surveyors were satisfied with the mobile form they filled out. In order to accomplish this, we administered post–field test interviews to all 33 surveyors.

### Ethical Considerations

This research paper reports retrospectively on prior interactions. The health clinic was run as part of the provision of social services, not specifically for this research project. The surveyors were volunteers with the clinic contracted for the purpose of administering surveys. All participants also had the option to withdraw at any time. Both of these activities were conducted in accordance with appropriate regulations, with all research procedures designed to align with the ethical principles outlined in the World Medical Association Declaration of Helsinki and adhered to relevant national and organizational standards for research involving human participants. The study reported in this paper made use of data that had already been collected in the course of normal operations, was anonymized, and finally provided to the researchers for analysis. Since this research was conducted on existing anonymized data, institutional review board approval and informed consent were not obtained specifically for this study.

## Results

### Pilot Interview Analysis

Our pilot survey asked several interview questions to better understand the target group. When asked what purpose surveyors used their phones for, surveyors indicated high use of social media (43/53, 81%) and messaging (39/53, 74%) apps on their phones, which may suggest a general level of comfort with mobile technology.

From our pilot interview, we learned that people had 2 concerns with mobile form surveys. First, a mobile form would be slower and more difficult to use than paper. Second, an unreliable network could render the form unusable. These 2 key concerns led us to creating our own custom survey forms software.

While we had anticipated the majority of surveyors preferring paper, only 40% (21/53) responded that they had a preference for paper, with the remaining 60% (32/53) preferring digital surveys. When asked why they preferred paper over digital surveys, the most common answer was that paper was “easier” or “faster” (17/21). Other answers included lack of phone ownership and concerns over poor internet access.

When observing how they typed, 32% (17/53) of surveyors typed with 1 finger, which may reflect a lack of experience and proficiency using modern digital phones. The rest typed with 2 fingers or used speed enhancements such as the autocomplete or swiping features on newer phones. The surveyors who typed with 1 finger preferred paper 53% of the time, while those using more advanced typing methods preferred paper 30% of the time. While we noticed a minor correlation between age and technical proficiency, as indicated by the typing method used, the data were insufficient to draw any definitive conclusions.

When asked if they had ever administered a population health survey before, more than 80% (43/53) responded no.

We concluded that many of the surveyors are not proficient modern phone users and have little experience in conducting health care surveys.

### Field Test Analysis

In order to see how our surveyors actually performed when using the mobile form, we analyzed factors related to speed of completion of survey, which we hypothesized might be related to their reluctance to use mobile forms.

Table 1 shows the average time taken to complete each question. Notice that on average, multiple choice questions took a shorter amount of time to answer. Several of the questions could have been framed as either text or multiple choice, and based on this finding, we will encourage the health team to use multiple choice when possible.

**Figure 4.**
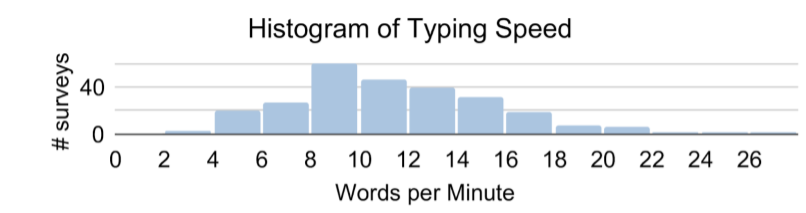
Our mobile survey is capable of analyzing surveyor’s typing speed. The average speed was 11.5 words per minute.

Average typing speed in textboxes across all 266 surveys was 11.5 words per minute; however, there was significant variation as shown in the histogram in Figure 4. This is consistent with previously reported typing speeds for low-resource populations [11].

**Table 1.** Average time to answer survey questions.^a^

Question	Type	Time (seconds)
Gender	Multiple choice	2.9
Type of phone owned?	Multiple choice	7.5
Source of clean drinking water?	Multiple choice	9.4
How do you cook?	Checkboxes	9.4
Lost wage for coming today?	Text	10.5
What do you do for work?	Text	11.9
How long does it take to go there?	Text	13.0
When did you last see a traditional healer?	Text	13.4
Cost to travel to a medical center?	Text	13.8
What is your education level, highest grade finished?	Text	14.5
About how much did it cost (roughly)? (Doctor’s Visit)	Text	15.8
When was your most recent visit to a doctor?	Multiple choice	16.6
Date of Birth	Text	17.5
Your name (the volunteer doing the survey)	Text	18.5
What is the closest medical center or clinic that you would go to?	Text	20.4
Type of condition patient seeking evaluation and treatment	Checkboxes	21.9

a After field-testing was completed, we analyzed the time it took to complete each question. These data will be used to revise the survey to minimize the time needed to collect information.

From the tracker that was built to record the time the internet was available while taking the survey, we found that 25% of the time the internet was unavailable during the survey. This indicates that a survey that can work offline is necessary.

### Post–Field Test Interview

We collected data on whether surveyors found the mobile form easy to use. When asked “How easy was the mobile phone survey to use?”, responses were very easy (n=23), somewhat easy (n=8), neither easy nor hard (n=2), somewhat hard (n=0), very hard (n=0). Of the 33 surveyors, 70% (n=23) agreed the mobile form was very easy to complete. No surveyors thought it was difficult to use. We also asked surveyors to describe positive attributes about mobile forms. A total of 30 out of 33 gave responses including the words “faster” or “easier,” in contrast to opinions prior to using the forms. Results from these questions illustrate the effectiveness of our mobile forms.

Another question was the preference for paper or mobile forms. In the pilot interviews, we found that 60% (32/53) of the surveyors preferred mobile forms. In the post–field test interviews, we asked surveyors if they were to conduct another survey, would they use paper or mobile forms. 76% (25/33) responded that they preferred mobile over paper. Since the percentage of surveyors preferring mobile forms increased after the field test, we hypothesize that actual experience of using mobile forms for surveying improved their opinions. Since the surveyors on average only completed the mobile form 8 times during the field test, we believed with more usage, preference for mobile forms would continue to increase. The majority of surveyors expressed a strong preference for mobile forms, based on their user experience. While this feedback is subjective, it highlights the potential for mobile forms to be well received in future applications, though additional objective performance metrics would strengthen the validation of these findings.

## Discussion

### Principal Findings

Large-scale population health surveys are essential to deploy health care resources efficiently. We investigated the feasibility of using mobile forms to conduct such surveys in a low-resource environment in the Philippines. Initially, field team organizers requested to use paper to conduct the survey since many of the volunteer surveyors asked for it and there was the concern of an intermittent network. However, pilot interviews revealed a mobile form was actually preferred by 60% (32/53) of the surveyors. Based on insights gleaned from the pilot interviews, we built survey forms software that generated mobile form surveys. We then ran field trials to test the mobile form and conducted interviews afterward. The health survey was successfully completed using the mobile form. The percentage of surveyors preferring mobile forms increased to 76% (25/33) after just using the form a few times. The results demonstrate our mobile form is a viable method to conduct large-scale population health surveys in this low-resource environment.

### Comparison to Prior Work

Several studies have already compared mobile-based surveys to paper-based data collection methods when it comes to health surveys. With an increase in the ownership of mobile phones, switching from paper surveys to digital surveys is becoming more appealing [12]. One study in Sudan found smartphone-based collection had fewer errors and faster retrieval than paper methods, seeing a reduction from an 83% error rate with paper questionnaires to a 17% error rate with smartphones [6]. In addition to that, data collected via smartphones were uploaded to the central database in a median time of 7 days, whereas paper-based data took a median of 21 days to be entered [13].

Our study builds on previous studies by developing a custom mobile form software, tested specifically in low-income environments in the Philippines. Our findings align with findings from a similar study done in rural Philippines using mobile apps [14]. Cost-effectiveness and reducing error rates have been focal points in recent studies, while implementing offline functionality has been studied less in recent studies. Our research advances this by developing custom mobile form software tailored for low-resource settings that is also extremely cost-efficient [15].

Unlike many studies that use existing platforms such as ODK, our custom mobile forms software offers a unique contribution by adding a focus on local surveyor needs for added equity when it comes to data collection. Our study further contributes to existing literature by providing insights into the implementation process of mobile forms in low-resource environments. The custom mobile forms software we developed addressed many of the common challenges identified in previous studies, such as the need for tools that can operate effectively in settings with limited or no internet connectivity [16].

Another example of an electronic data capture (EDC) framework designed for low-resource environments is ConnEDCt (Weill Cornell Medicine), a mobile EDC platform developed specifically for clinical research applications in India and Ecuador. While their system also supported offline data collection and synchronization, their system was designed for more complex clinical research protocols including randomized controlled trials and regulatory-compliant data handling; however, our system focuses on a more simple and scalable approach for health surveys that can be set up quickly [17].

While there are a large range of tools in use, many do not offer a complete set of features and often require users to use multiple tools in parallel, thus complicating the workflows [7]. Our system fixes this issue as we consolidate every feature needed in a low-resource environment into one system in order to streamline the workflow.

### Limitations

The study had several limitations that could influence the results of the study and its interpretation. First, the geographic scope of the study was restricted to Metro Manila, which limited the generalizability of the findings to other regions that could have different socioeconomic and technological conditions. Future studies could broaden the geographic scope to include more representation. Second, the sample size of the surveyors was small and future studies could use a larger and more diverse set of participants. To mitigate this limitation, we conducted pilot interviews to gather initial feedback, though future research should aim for larger scale studies to capture any potential variability. Third, the involvement of surveyors in testing the software may have introduced bias. While we tried to minimize biases by anonymizing survey responses and emphasizing the importance of honest feedback, future studies should consider using an independent group of testers. Fourth, technological familiarity among the surveyors, particularly the use of mobile phones, varied widely, potentially also affecting the usability testing. During our pilot interviews, we included questions that aimed to gauge the surveyors’ comfort and proficiency with technology; however, we acknowledge that this initial assessment was not a comprehensive measurement of the surveyor’s general literacy skills. Future studies should incorporate a more comprehensive evaluation of general literacy levels.

## Abbreviations

EDC: electronic data capture
